# Impact of Fishmeal Replacement in Diets for Gilthead Sea Bream (*Sparus aurata*) on the Gastrointestinal Microbiota Determined by Pyrosequencing the 16S rRNA Gene

**DOI:** 10.1371/journal.pone.0136389

**Published:** 2015-08-28

**Authors:** G. Estruch, M. C. Collado, D. S. Peñaranda, A. Tomás Vidal, M. Jover Cerdá, G. Pérez Martínez, S. Martinez-Llorens

**Affiliations:** 1 Aquaculture and Biodiversity Research Group. Institute of Science and Animal Technology, (ICTA), Universitat Politècnica de València, Valencia (Valencia), Spain; 2 Department of Biotechnology, Institute of Agrochemistry and Food Technology, Spanish National Research Council (IATA-CSIC), Paterna (Valencia), Spain; Field Museum of Natural History, UNITED STATES

## Abstract

Recent studies have demonstrated the impact of diet on microbiota composition, but the essential need for the optimization of production rates and costs forces farms and aquaculture production to carry out continuous dietary tests. In order to understand the effect of total fishmeal replacement by vegetable-based feed in the sea bream (*Sparus aurata*), the microbial composition of the stomach, foregut, midgut and hindgut was analysed using high-throughput 16S rDNA sequencing, also considering parameters of growth, survival and nutrient utilisation indices.A total of 91,539 16S rRNA filtered-sequences were analysed, with an average number of 3661.56 taxonomically assigned, high-quality sequences per sample. The dominant phyla throughout the whole gastrointestinal tract were *Actinobacteria*, *Protebacteria* and *Firmicutes*. A lower diversity in the stomach in comparison to the other intestinal sections was observed. The microbial composition of the Recirculating Aquaculture System was totally different to that of the sea bream gastrointestinal tract. Total fishmeal replacement had an important impact on microbial profiles but not on diversity. *Streptococcus (p-value*: *0*.*043) and Photobacterium (p-value*: *0*.*025)* were highly represented in fish fed with fishmeal and vegetable-meal diets, respectively. In the stomach samples with the vegetable diet, reads of chloroplasts and mitochondria from vegetable dietary ingredients were rather abundant. Principal Coordinate Analysis showed a clear differentiation between diets in the microbiota present in the gut, supporting the presence of specific bacterial consortia associated with the diet.Although differences in growth and nutritive parameters were not observed, a negative effect of the vegetable diet on the survival rate was determined. Further studies are required to shed more light on the relationship between the immune system and sea bream gastrointestinal tract microbiota and should consider the modulation of the microbiota to improve the survival rate and nutritive efficacy when using plant-based diets.

## Introduction

The gilthead sea bream (*Sparus aurata*) is a species of the family Sparidae being produced in large amounts in Europe. As a carnivorous fish, it requires a high level of fishmeal in its diets to provide an ideal amino acid profile and reach high digestibility and growth. Despite this, fishmeal substitution by plant protein sources in sea bream diets is necessary to maintain the profitability of the farms. Therefore, in recent years, a large research effort has been made in this field to reduce fishmeal and/or fish oil in aquafeeds by plant sources [1, 2]. However, plant protein sources contain certain undigestible components (non-starch polysaccharides) [3] and antinutritional factors (protease inhibitors, lectins, phytic acid, saponins, phytoestrogens, antivitamins, allergens) [4]. These compounds can affect nutrient digestibility and absorption [5], as well as gut integrity [6,7], promoting bacteria ingress and, therefore, change the gut microbiota in terms of microbial abundance and species richness.

Despite these problems associated to vegetable proteins, a successful replacement of total fishmeal by a vegetable protein concentrate mixture has been reported [8]. However, alterations in the gut histology of sea bream have been observed with fishmeal replacement above a 60% level [9], as well as immunosuppression above 75% of fishmeal substitution [6]. An imbalanced microbiota may provoke an alteration of the immune regulatory functions of the gut and contribute to the development of diseases [10].

Many exogenous and endogenous factors, such as species, age and developmental stage, bacterial colonisation during the larval stage, geographic location, seasonality and other environmental factors, especially temperature, antibiotic use during fish growth, or the individual genetics of each fish can alter the gut microbiota composition [11]. However, food is one of the main factors putting selective pressure on the gastrointestinal microbial composition [12]. Differences in the amount of the microbiota population between fish fed live food or artificial feed have been observed [13]. Also, diets with plant meals have an impact on the microbiota composition [14], affecting the gastrointestinal tract (GIT) morphology and increasing the damage of the absorptive area [15], although differences in microbiota composition were not observed in herbivorous fish species such as the Crucian Carp (*Carassius auratus gibelio* x *Cyprinus carpio*) [16].

It is frequently considered that fish gut is usually divided into three sections [17, 18]: the first segment or foregut (FG) is generally the longest part and has mainly an absorptive function, the second segment or midgut (MG) contains enterocytes having a high pinocytotic activity for macromolecule transport, and the third segment or hindgut (HG) is the shortest of them, for which different functions have been proposed. Different digestive functions may also be related to different microbial content as occurs in mammals [19]. Furthermore, although the gut associated lymphoid tissue (GALT) [10] in fish does not reach the level of organisation shown in mammals, abundant lymphocytes are found in the lamina propria. The midgut has been proposed as the segment that has a clearer function of antigen capture and immune stimulation [20], and also a high presence of immunoreactive cells has been associated to the posterior intestine [21].

Fish microbiota has traditionally been studied by culture methods and subsequent identification based on biochemical and phenotypic characteristics of bacteria [22]. The development of PCR-DGGE (Polymerase Chain Reaction—Denaturing Gradient Gel Electrophoresis) [23] and other molecular methods in recent years has allowed the characterisation of total microbiota in fish, both marine fish and freshwater, such as *Oncorhynchus mykiss* [24], *Gadus morhua* [14], *Salmo salar* [25], *Paralichthys olivaceus* [12] and many other species, including *Sparus aurata* [7,15,26]. However, new modern sequencing techniques such as 454 pyrosequencing (Roche, Basel, Switzerland) have been applied to study the microbiota of zebrafish (*Danio rerio*) [27], and also economically important species such as *Cyprinus carpio* [28], *Oncorhynchus mykiss* [29], *Dicentrarchus labrax* [30] or *Sparus aurata* [31].

The aim of this study was to assess the impact of a total vegetable diet during the fattening period of sea bream on zootechnical parameters, but also on an increasingly relevant biological aspect, the gut microbiota composition, that may in turn have a number of physiological consequences ranging from feed component utilisation to immune competence. Furthermore, to the best of our knowledge, this study represents the first report of microbiota composition along the GIT in sparids fed only with vegetable meals as a source of protein using high-throughput techniques.

## Materials and Methods

### Rearing system

The trial lasted 154 days (from December 2012 to May 2013) and was conducted in six cylindrical fibre glass tanks (1750 L) as part of a recirculating saltwater system (75 m^3^ capacity) with a rotary mechanical filter and a 6 m^3^ capacity gravity biofilter. All tanks were equipped with aeration, and the water was heated with a heat pump installed in the system. The water temperature was 22.0±0.52°C, salinity was 30±1.7 g L^-1^, dissolved oxygen was 6.5 ± 0.49 mg L^-1^, and pH ranged from 7.5 to 8.5. The photoperiod was natural and all tanks had similar lighting conditions.

### Fish

Sea bream were obtained from the fish farm PISCIMAR in Burriana (Valencia, Spain) and after two months of acclimation to laboratory conditions, feeding a standard commercial diet, were distributed in the six tanks in groups of 20 in each tank. The experiment was initiated with fish weighing 130 ± 19 g, however, with slight differences between the tanks.

### Ethics statements

The experimental protocol was reviewed and approved by the Committee of Ethics and Animal Welfare of the Universitat Politècnica de València (UPV), following the Spanish Royal Decree 53/2013 on the protection of animals used for scientific purposes [32].

### Diets and feeding

Diets were prepared as pellets by cooking-extrusion with a semi-industrial twin-screw extruder (CLEXTRAL BC-45, Firminy, St Etienne, France); located at UPV. The processing conditions were as follows: 0.63 g screw speed, 110°C and 30–40 atm. Proximate analyses of diet ingredients, diets and faeces were based on AOAC procedures [33].

Two isonitrogenous and isoenergetic diets (FM100 and AA0) were formulated using commercial ingredients (Table 1). FM100 contained fishmeal as the main protein source, wheat meal, fish and soy oil and a vitamin-mineral mix. In the AA0 diet, fishmeal and wheat meal were replaced by a mixture of vegetable meals, and synthetic aminoacids were added in order to balance the aminoacid composition. Proximate composition, including digestible protein (DP), is also shown in Table 1. Apparent digestibility of the protein of feeds was determined using the method detailed by Sánchez Lozano et al. [34].

**Table 1:** Ingredient content and proximate composition of experimental diets.

	**FM100**	**AA0**
**Ingredients (g kg** ^**-1**^ **)**		
**Fishmeal**	589	
**Wheat meal**	260	
**Wheat gluten**		295
**Bean meal**		41
**Soybean meal**		182
**Pea meal**		41
**Sunflower meal**		158
**Fish oil**	38,1	90
**Soybean oil**	92,9	90
**Soy Lecithin**	10	10
**Vitamin-mineral mix** *	10	10
**Calcium phosphate**		38
**Taurine**		20
**Methionine**		7
**Lysine**		10
**Arginine**		5
**Threonine**		3
**Proximate composition (% dry weight)**		
**Dry matter**	88	94
**Ash**	10,1	7,4
**Crude lipid (CL)**	18,5	19,8
**Fibre**	1	4,2
**NFE** **	26	22,2
**Non-starch polysaccharides**	10,9	20,6
**Protein (CP)**	44.2	45.0
**Digestible Protein (DP)**	42,4	41,4

*Vitamin and mineral mix (values are g kg^− 1^ except those in parenthesis): Premix: 25; Choline, 10; DL-a-tocopherol, 5; ascorbic acid, 5; (PO_4_)_2_Ca_3_, 5. Premix composition: retinol acetate, 1 000 000 IU kg^− 1^; calcipherol, 500 IU kg^− 1^; DL-a-tocopherol, 10; menadione sodium bisulphite, 0.8; thiamine hydrochloride, 2.3; riboflavin, 2.3; pyridoxine hydrochloride, 15; cyanocobalamine, 25; nicotinamide, 15; pantothenic acid, 6; folic acid, 0.65; biotin, 0.07; ascorbic acid, 75; inositol, 15; betaine, 100; polypeptides 12.

**Nitrogen free extract (NFE, %) = 100—%CP—%CL—%Fibre

### Growth assay

Each experimental diet was assayed along 154 days in three tanks, randomly assigned. Fish were handfed twice a day (09:00 and 17:00 hours) to apparent satiation in a weekly feeding regimen of six days and one of fasting. Pellets were distributed slowly, permitting all fish to eat. Fish were observed daily in tanks and were weighed individually every four weeks, using clove oil containing 87% eugenol (Guinama, Valencia, Spain) as an anaesthetic (1 mg / 100 mL of water) to minimize their suffering, in order to evaluate fish growth along the assay, determine growth parameters and assess their health status. Growth and nutrient utilisation indices considered were as follows:

Specific growth rate (% day^-1^) (SGR) = 100 ln (final weight / initial weight) / days

Feed intake (g 100 g fish^-1^ day^-1^) (FI) = 100 feed consumption (g) / (average biomass (g) days)

Feed conversion ratio (FCR) = feed offered (g) / weight gain (g)

Protein efficiency ratio (PER) = weight gain (g) / protein offered (g)

Survival (%) (S) = 100 (final number of fish / initial number of fish)

### Sampling of gastrointestinal contents

Gastrointestinal contents of three fish per tank were sampled at the end of the assay in the laboratory, 154 days after initiation of the experiment. The fish were slaughtered to obtain samples of the gastrointestinal content in the different sections of the GIT.

The criterion used to determine when the animals should be humanely sacrificed was their commercial size (over 300 g). To ensure the presence of content along the whole digestive tract, fish were fed at 20:30 on the day before and at 8:30 on the sampling day, 30 minutes before initiation of sampling. Samples of intestinal contents would originate from the previous meal and not from the food ingested just 30 minutes before, cause in seabream, food remains in the stomach for 6 hours, approximately.

Fish were anesthetized using clove oil dissolved in water (1 mg / 100 mL of water), in order to minimize suffering of the animals,sacrificed by decapitation, and then dissected in order to obtain the digestive tract. Four different sections were considered: stomach (ST), foregut (FG), midgut (MG) and hindgut (HG). Gastrointestinal content was obtained by scrapping the gastric/intestinal mucosa with a spatula, whereby samples include the luminal and the mucosa-associated microbiota. Thus, a total of four gastrointestinal content samples were obtained per fish, placed in Eppendorff tubes and immediately frozen in liquid nitrogen. Later, they were stored at -80°C until DNA extraction. Moreover, a 500 mL water sample was obtained from the recirculating saltwater system and stored at -20°C.

72 samples of gastrointestinal content were obtained in the sampling. Nevertheless, samples were pooled after 16S ribosomal RNA gene PCR amplification to simplify the pyrosequencing assay and subsequent microbiota analysis. Each pool was made up of 3 samples from the same gastrointestinal section, proceeding from the same tank. Hence, each pool represents a particular digestive section of a single tank, having a total of 24 pools. The water sample was assayed simultaneously; i. e. 25 different sets of sequences were obtained from the raw pyrosequencing data.

### DNA extraction

Total DNA was isolated from the gastrointestinal content samples by using the Genomic DNA from tissue Kit (Macherey-Nagel, Germany), according to the manufacturer’s instructions.

### PCR amplification and pyrosequencing

A barcoded primer set based on universal primers 27F and 533R was used to amplify 500 bps of the 16S rRNA genes covering the V1 to V3 regions. PCR was carried out using a high-fidelity KAPA-HiFi polymerase (Kappa Biosystems, US) with an annealing temperature of 52°C and 30 cycles to minimise PCR biases. All samples assigned to the same pool shared a common barcode. The final DNA per sample was measured using the Agilent High Sensitivity DNA assay in the Agilent 2100 Expert.

Purified PCR products were pooled in equimolar amounts, as described in the 454 Roche protocol, and submitted for pyrosequencing, using the Genome Sequencer GS Junior Series (454 Life Science, Branford, USA). PCR products were pyrosequenced from the forward primer end only at Servei Central de Suport a la Investigació Experimental (SCSIE) of the Universitat de València (Valencia, Spain).

### Livestock data statistical analysis

Statistical data analyses were carried out with Statgraphics Centurion XVI [35]. SGR, FCR, FI, PER and S data were subjected to multifactor variance analysis, introducing the initial live weight as a covariate in growth data. Each group in the calculation represented the combined group of fish per single tank (triplicate tanks per treatment). The Newman—Keuls test was used to assess specific differences among diets at the 0.05 significance level. Descriptive statistics are mean ± SE unless otherwise noted.

### Sequence data analysis

From the resulting raw data set, provided by pyrosequencing, low quality sequences were filtered out to remove sequences having a length shorter than 150 nucleotides. A dereplicate request on the QIIME pipeline was used to identify representative sequences for each operational taxonomic unit (OTU) generated from complete linkage clustering with a 97% similarity, and chimeric sequences were removed using UCHIME software [36]. Reads of chloroplast, mitochondria or eukaryotic origin were also excluded by filtering sequences. Alpha diversity indices were determined from rarefied tables using the Shannon-Wiener index for diversity and the Chao1 index for species richness; Observed Species (number of unique OTUs) and Phylogenetic Distance (PD_whole) were also determined. A beta diversity distance matrix was computed from the previously constructed OTU table using UniFrac analysis. Unweighted (presence/absence matrix) and weighted (presence/absence/abundance matrix) UniFrac distances were used to construct two- and three-dimensional Principal Coordinate Analysis (PCoA) plots. Biplots were generated as part of the beta diversity analysis in QIIME, using genus level OTU tables showing principle coordinate sample clustering alongside weighted taxonomic group data. Data on assigned sequences at genus level shared between samples were used to generate a Venn diagram.

Relative frequencies of different taxonomic categories were calculated using the Statistical Analysis of Metagenomic Profiles program (STAMP v.2.0.0). Statistical differences between experimental fish samples were estimated by ANOVA analysis with the Games-Howell post-hoc test and the multiple test correction of Benjamini-Hochberg and differences between diets were calculated by T-test as implemented in STAMP.

DNA sequences were deposited in the MG-RAST server database (http://metagenomics.anl.gov/, with access numbers 4548816.3 to 4548840.3), under the project name “Seabream Gastrointestinal Microbiota.”

## Results

### Performance factors of gilthead sea bream

No differences were found in growth and nutritive parameters (Table 2) between fish fed two diets during the on-growing phase. However, fish survival showed significant differences, and the FM100 diet presented a higher survival rate (88%) than the AA0 diet (60%).

**Table 2:** Main performance of gilthead sea bream fed diet FM100 or AA0. Means of triplicate groups. Data in the same row with different superscripts differ at P<0.05. SME: pooled standard error of the mean. Specific growth rate (%day−1), **SGR** = 100×ln(final weight/initial weight)/days. Feed Intake ratio (g 100 g fish−1day−1), FI = 100×feed consumption (g)/average biomass (g)×days. Feed Conversion Ratio, FCR = feed offered (g)/ weight gain (g). Protein Efficiency Ratio PER = Weight gain (g)/Protein intake (g). Initial average weight: FM100; Tank 1: 133±18.9, Tank 2: 136±23.7, Tank 3: 125±16.0. AA0; Tank 1: 129±21.2, Tank 2: 127±15.1, Tank 3: 126±17.0.

	FM100	AA0	SEM
**Final weight (g)**	393	360	15.7
**Survival (%)**	88^a^	60^b^	5.5
**SGR (% day** ^**−1**^ **)**	0.72	0.69	0.040
**FI (g 100 g fish** ^**−1**^ **day** ^**−1**^ **)**	1.35	1.38	0.019
**FCR**	2.14	2.40	0.122
**PER**	1.06	0.96	0.063

### Gut microbiota composition of gilthead sea bream

**Fig 1:**
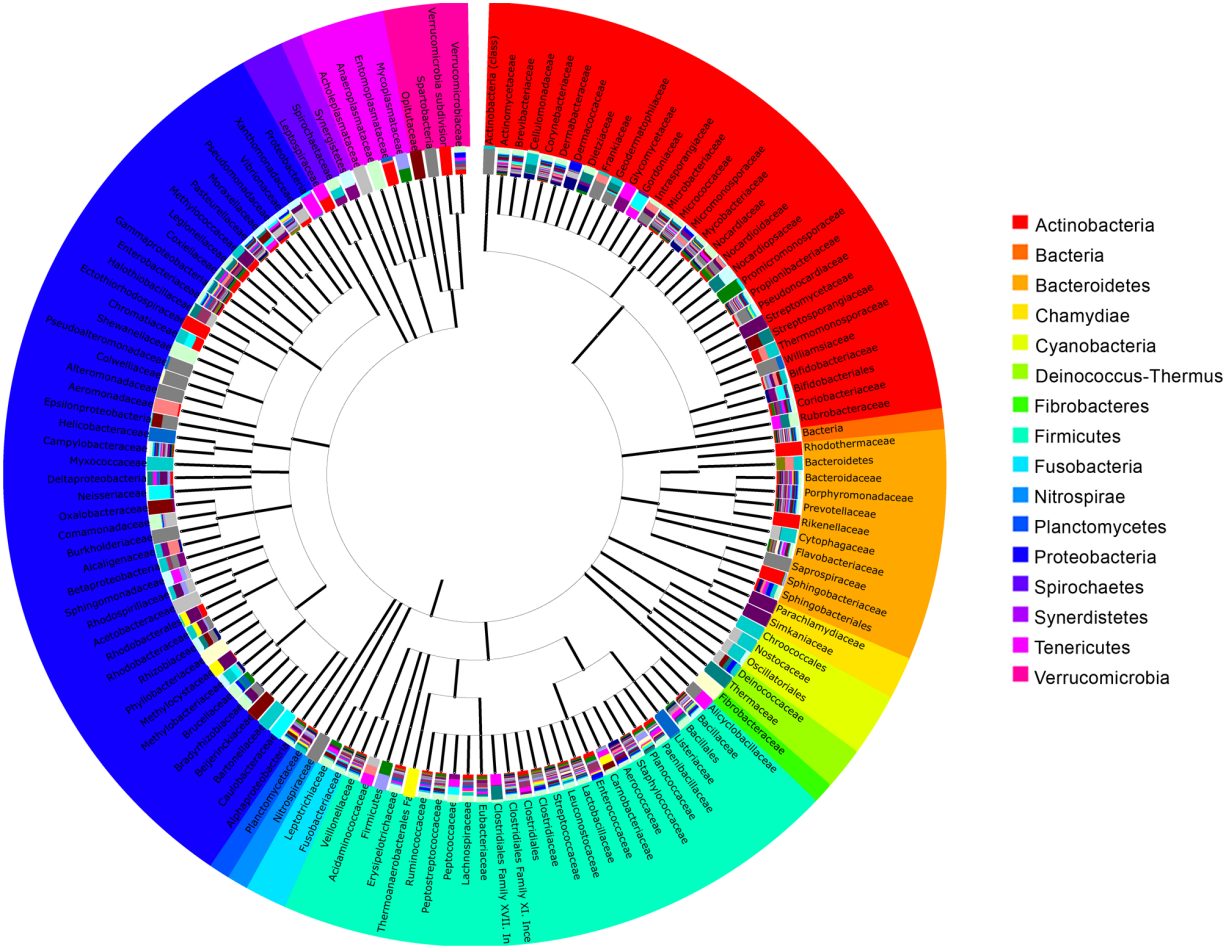
Circular tree representation of microbiota associated to the GIT of the gilthead sea bream, at family taxonomic level. Sequences were assigned to different families using GreenGenes database in MG-RAST, with a minimum percentage of identity cutoff of 80% and a minimum alignment length cutoff of 15. Different colours were assigned by phyla. Likely chloroplast and mitochondria sequences were omitted. Amount of different colours in the bars are representative of the number of different MIDs in which each taxon was found. Phylogenetic relations between different taxa are shown.

After quality filtering and length trimming, 91,539 16S rDNA sequences were analysed, with an average number of 3661.56 taxonomically assigned, high-quality sequences per sample. The microbiota throughout the GIT of gilthead sea bream was analysed and sequences annotated in OTUs with the QIIME pipeline using the GreenGenes database. A total of 2,813 de novo OTUs at 97% identity were identified in gilthead seabream GIT. A total of 43,177 sequences (4,793 hits, with an average percentage of identity of 99.16) could be identified at species level, 56,400 (7,026 hits) at genera level and 70,721 (7388 hits) at family level. Families grouped schematically as shown in Fig 1 using a minimum identity cut-off of 80%.

**Fig 2:**
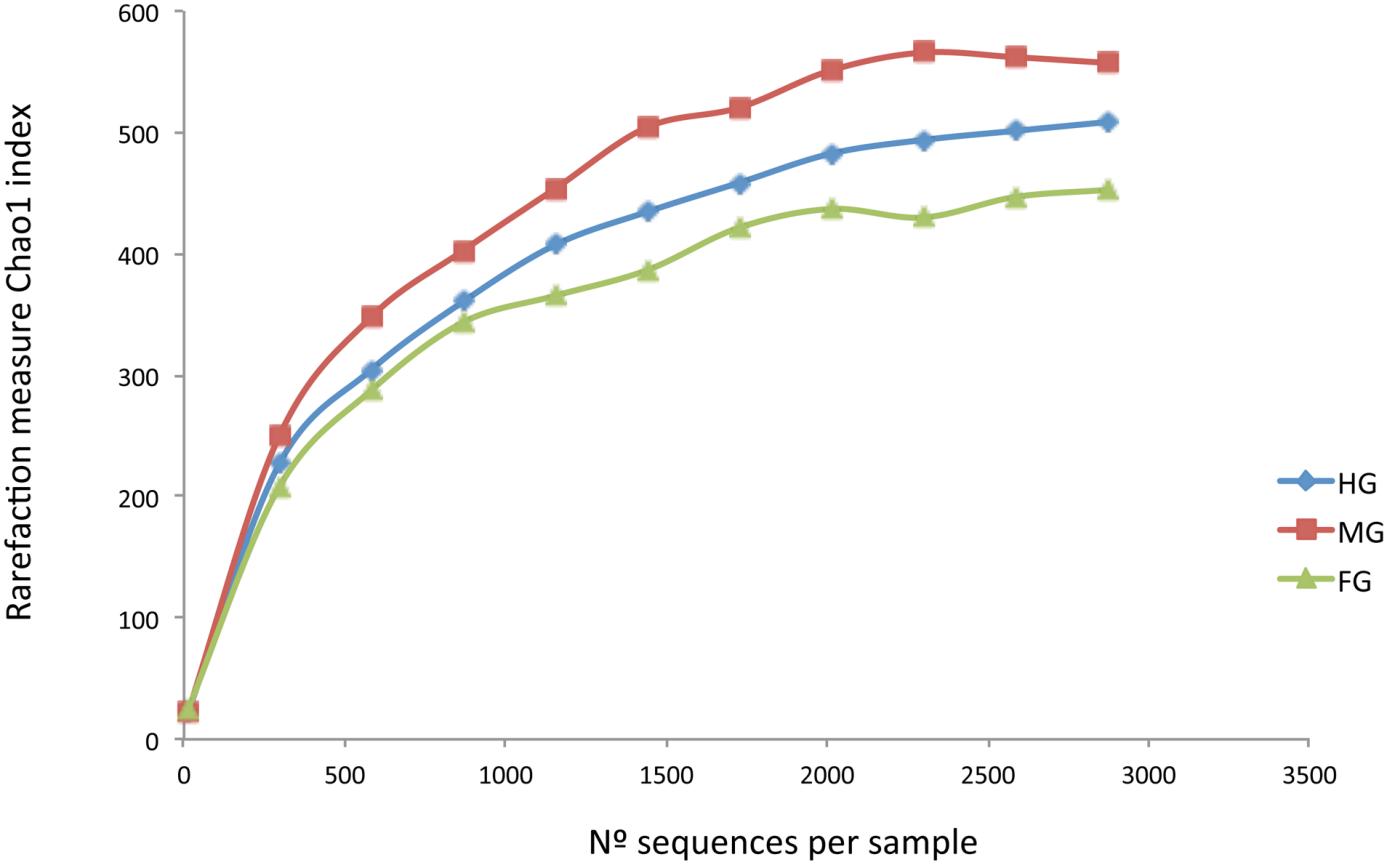
Rarefaction curves (Chao1 index) showing the microbial community complexes in the different gut sections of the gilthead sea bream. Orange = FG; Blue = MG; Red = HG.

When analysing separately sequences from different segments of the GIT from all the animals analysed, a lower number of sequences was found for the ST in comparison with the gut sections. No significant differences in species richness (Chao1 index) among different gut sections (FG, MG, HG) could be found when a rarefaction analysis was performed (Fig 2).

The dominant phyla in ST were Firmicutes (29.1%), *Proteobacteria* (26.0%), and *Actinobacteria* (24.8%). In the FG, the predominant phyla were *Actinobacteria* (34.8%) and *Firmicutes* (33.0%), followed by *Proteobacteria* (24.8%). *Firmicutes* was the dominant phylum in the MG (33.7%) and *Actinobacteria* and *Proteobacteria* exhibited similar percentages (27.6% and 25.6%, respectively). Finally, Actinobacteria was the most represented phylum in the HG (36.5%), followed by *Proteobacteria* (31.7%), while *Firmicutes* was less abundant (23.7%) in comparison with other sections of the GIT. Another 21 phyla were found along the whole digestive tract, including *Bacteroidetes* (from 3.6% to 6.7%). A large percentage of unassigned bacteria was also found ranging from 2.8% (in the FG and the MG) to 13.0% (in the ST).

Although all the *Cyanobacteria* sequences, including sequences assigned to the *Chloroplast class*, were removed from the analysis, some non-plant derived sequences belonging to the*4C0d*-2; *ML635J-21*; *Nostocophycideae* and *Oscillatoriophycideae* classes, (of the families *Xenococcaceae* and *Phormidiaceae*), related to marine algae, were also found.

Performing the analysis at genus taxonomic level, *Streptococcus* (7.8%) and *Clostridium* (7.2%) were the most abundant genera among *Firmicutes*. Regarding *Actinobacteria*, the genus *Corynebacterium* was predominant along the whole digestive tract, including the ST (11.5%), followed by the genus *Propionibacterium* (4.5%). Families of the phylum Proteobacteria highly observed in the stomach were *Vibrionaceae* (11.3%), mainly from the genus *Photobacterium* (8.4%), and *Enterobacteriaceae* (3.6%). The percentage of unassigned sequences in this section was remarkable (13.7%).

Different genera of *Proteobacteria* were found in the FG in higher proportions, such as *Photobacterium* (4.4%), *Enhydrobacter* (3.7%), an unassigned genus (UG) of the family *Enterobacteriaceae* (3.7%) and *Sphingomonas* (2.7%). In relation to *Actinobacteria*, genera *Corynebacterium* and *Propionibacterium* were found in higher amounts than in the ST (20.3% vs 11.5%, p-value = 0.243, and 8.4% vs 4.5%, p-value = 0.085, respectively). Among *Firmicutes*, *Streptococcus* was the most abundant genus (14.1%), followed by *Staphylococcus* (2.7%), *Finegoldia* (2.4%) and *Lactobacillus* (1.9%), while the genus *Clostridium* decreased in the FG when compared to the ST (0.1% vs 7.2%, p-value = 0.090).

In the MG, *Firmicutes* was the most abundant phylum, dominated by *Lactobacillus* (7.1%), *Streptococcus* (6.6%), *Proteiniclasticum* (3.8%), *Megamonas* (2.8%), *Staphylococcus* (2.2%) and *Finegoldia* (1.6%). The phylum *Actinobacteria* was represented mainly by the genera *Corynebacterium* (13.4%) and *Propionibacterium* (11.5%). Regarding *Proteobacteria*, major changes with respect to the FG were not observed. *Photobacterium* (4.4%) and an UG of the family *Enterobacteriaceae* (3.8%) were observed, as well as *Pseudomonas* (3.6%) and other unassigned genera at this level belonging to the families *Legionellaceae* (2.3%) and *Rhodobacteraceae* (2.1%). The genus *Bacteroides* was relatively abundant in this section (4.5%).

Finally, an UG of the family *Micrococcaceae* was well-represented (4.5%) among genera of *Actinobacteria*, but *Corynebacterium* (17.1%) and *Propionibacterium* (10.4%) were still observed as the most abundant genera belonging to this phylum. The genus *Photobacterium* was highly represented in this section (11.5%), and other observed genera were the above-mentioned UG of families *Rhodobacteraceae* (4.1%), and *Legionellaceae* (4.1%). Regarding *Firmicutes*, *Streptococcus* exhibited a similar percentage when compared to the MG (8.2%), and the same genera identified in previous sections were found, such as *Lactobacillus* (2.0%), *Staphylococcus* (1.7%) and *Finegoldia* (1.4%).

### Recirculating Saltwater System Microbiota

**Fig 3:**
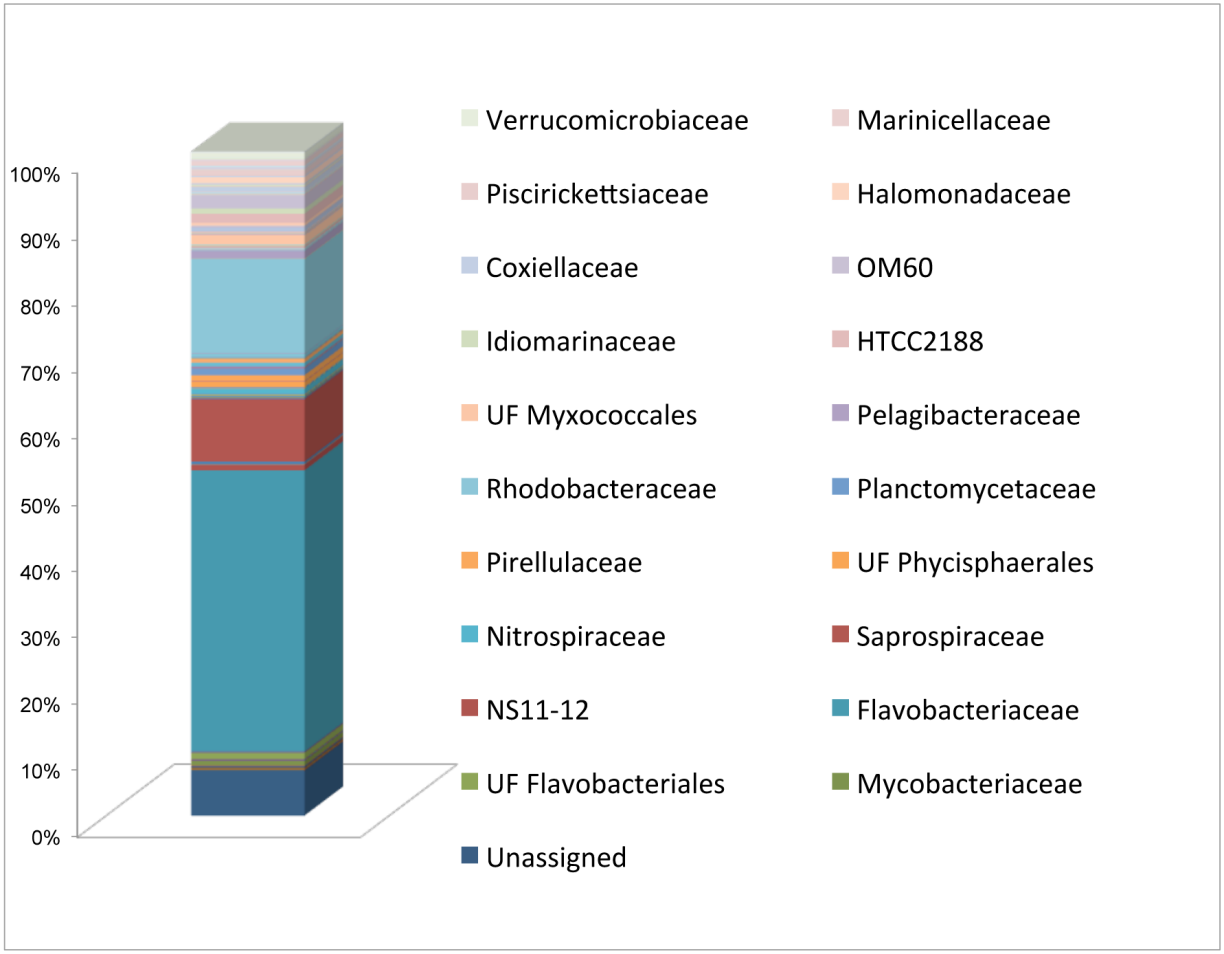
Relative abundance (%) of main taxa present in the water of the RAS, at family taxonomic level. Families with abundance lower than 0,5% in all samples were not shown.

In order to evaluate the mutual impact of the recirculating saltwater system and sea bream GIT, the microbiota present in the water was also analysed. *Bacteroidetes* (54.5%) was the predominant phylum, with *Flavobacteriaceae* (42.4%) as the most common family, and *Sediminicola* being the most represented genus (25.0%). *Saprospiraceae* were also identified (9.4%). The other predominant phyla were *Proteobacteria* (31.1%), including the family *Rhodobacteraceae* (14.2%), and *Planctomycetes* (3.08%), while the percentage of the phyla *Actinobacteria* and *Firmicutes* were lower than 1.5%, in contrast with those levels observed in sea bream GIT. Another 13 phyla were observed. Hence, dominant families observed in the gastrointestinal microbiota were significantly different from those observed in the water (Fig 3).

### Impact of fishmeal replacement on gut microbiota composition of gilthead sea bream

**Fig 4:**
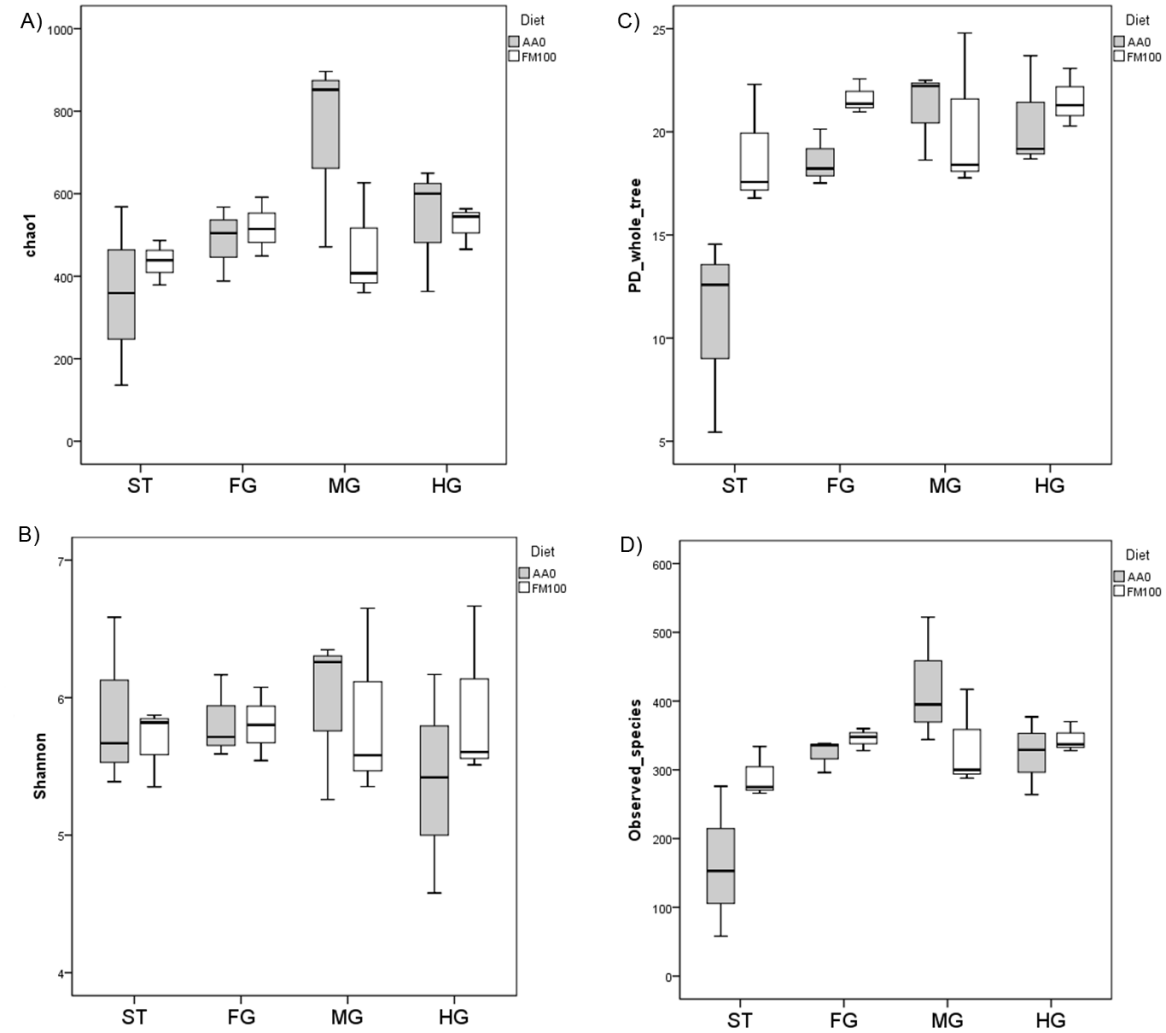
Alpha diversity metrics, Phylogenetic Distance and Observed Species throughout the GIT of the gilthead sea bream. A) Chao1 index (chao1); B) Shannon-Wiener index (Shannon); C) Phylogenetic distances (PD_whole_tree); D) Observed species (Observed_species). Different indices were represented by Box-Whisker diagrams for the two groups of fish and significant differences are indicated with an *.

Alpha diversity metrics (Chao1 and Shannon-Wiener indices) did not show differences between diets throughout the GIT. No significant differences were observed in Phylogenetic Distance or the Number of Species when fish fed the fishmeal diet were compared to fish fed the vegetable mixture diet (Fig 4). Rarefaction curves were shown in S1 Fig.

**Fig 5:**
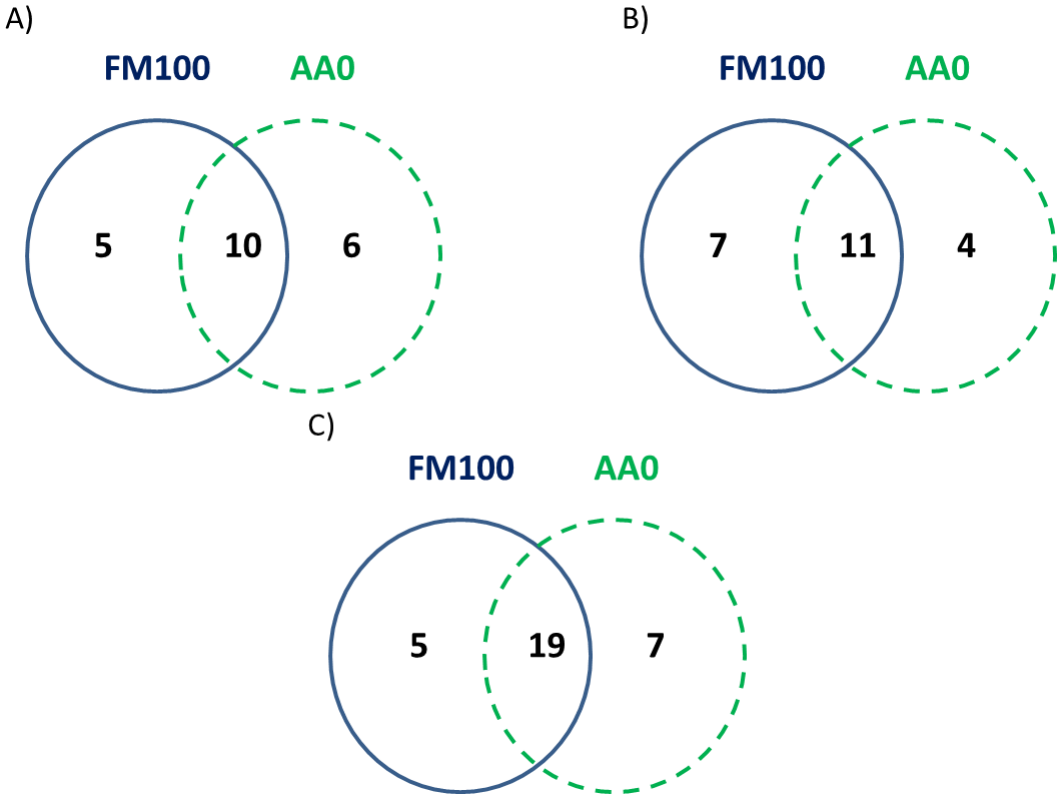
Venn diagrams for the different gut sections, at family taxonomic level. OTUs included were present in percentages above 1%. Common bacterial families are displayed in the middle regions and specific bacterial families of fish fed the AA0 and the FM100 diet are displayed in green and blue line colourd, respectively. (A) FG; (B) MG; (C) HG.

The Venn diagram showed a broader perspective (Fig 5). A core of 10, 11 and 19 bacterial families was shared by the two groups of fish in the FG, MG and HG, respectively. A greater number of families specifically associated with the diet FM100 was found in the MG (7 vs. 4 linked to the AA0 group), while fishmeal replacement slightly increased the number of specific genera in the FG (5 vs 6) and HG (5 vs 7). In the FG, Actinomycetaceae, Carnobacteriaceae, Micrococcaceae, Neisseriaceae and Pasteurellaceae families were also exclusive of fish fed FM100, while Methylobacteriaceae, Moraxellaceae, Porphyromonadaceae, Pseudomonadaceae, Shingomonoadaceae and Vibrionaceae were only observed in fish fed AA0. In the MG, the unique families in the FM100 group were *Bacteroidaceae*, *Coxiellaceae*, *Enterobacteriaceae*; *Lachnospiraceae*; *Legionellaceae*, *Pasteurellaceae* and *Ruminococcaceae*, whilst *Acetobacteraceae*, *Clostridiaceae*, *Leuconostocaceae* and Vibrionaceae were only found in the AA0 group. Finally, in the HG, *Actinomycetaceae*, *Coxiellaceae*, *Micrococcaceae*, *Pasteurellaceae* and *Rhizobiaceae* families were exclusively present in fish fed FM100; in contrast, *Clostridiaceae*, *Comamonadaceae*, *Enterococcaceae*, *Moraxellaceae*, *Nocardiopsaceae*, *Pseudomonadaceae* and *Vibrionaceae* were solely observed when fish were fed AA0.

**Fig 6:**
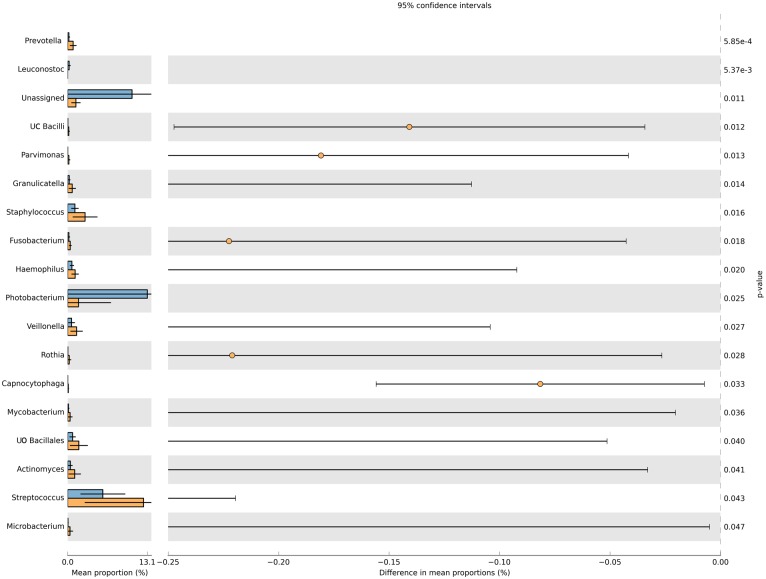
Significant differences between diets at genus level, independent of the gut section. Mean proportions, 95% confidence intervals and p-values are represented for each taxon for the two groups of fish. T-tests were used when comparing the relative abundances of individual taxa between AA0 and FM100.

Significant differences at genus level were found among diets when all the sections were considered (Fig 6), highlighting *Photobacterium* (p-value: 0.025) and *Streptococcus* (p-value: 0.043), which were highly represented in the AA0 and FM100 diets, respectively. Moreover, the AA0 diet exhibited higher relative percentages of unassigned sequences (p-value: 0.011).

**Fig 7:**
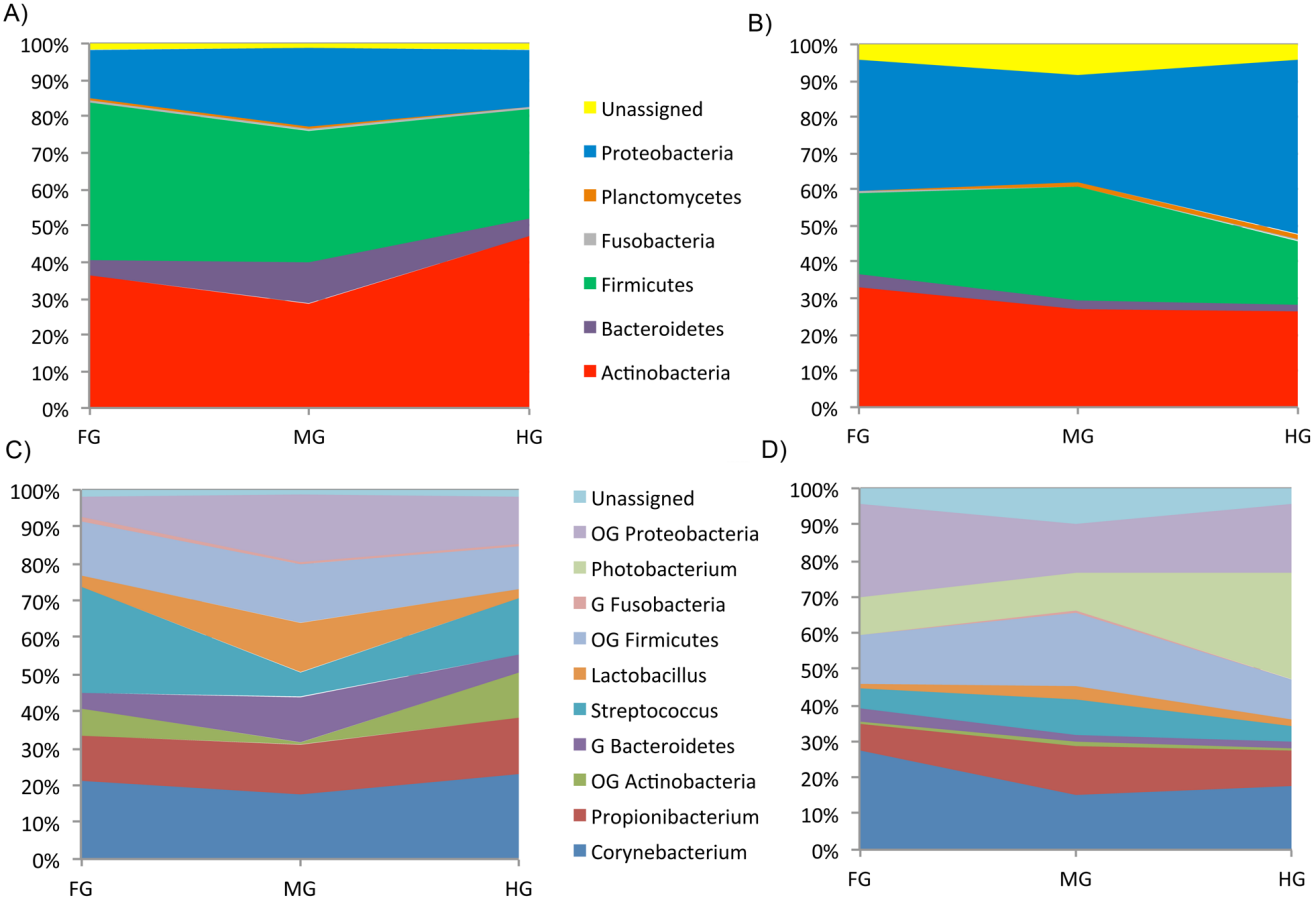
Relative abundance (%) of the main taxa present throughout the gut of the gilthead sea bream, according to the diet, at phylum and genus taxonomic level. Different sections (FG, MG, HG) are displayed in the X axis; relative abundance of different taxons are represented in the Y axis. Only phyla or genera with abundance higher than 0,5% in any gut section were shown. Only genera with abundance higher than 10% in any gut section were shown separately (G = genera; OG = other genera)*. A) Microbial community of fish fed FM 100, at phylum taxonomic level B) Microbial community of fish fed AA0, at phylum taxonomic level C) Microbial community of fish fed FM100, at genus taxonomic level D) Microbial community of fish fed AA0, at genus taxonomic level. * **OG Proteobaceria**: Enhydrobacter, UG Enterobacteriaceae, Haemophilus, UG Legionellaceae, Neisseria, Pseudomonas, UG Rhodobacteraceae and Shingomonas; **OG Firmicutes**: Delftia, Finegoldia, Granulicatella, Megamonas, Peptoniphilus, Proteiniclasticum, Staphylococcus and Veillonella; **G Bacteroidetes**: Bacteroides, Paraprevotella, Porphyromonas and Prevotella; **OG Actinobacteria**: Actinomyces, Microbacterium and UG Micrococcaceae.

Fig 7 represents the evolution of the main taxa along the intestinal tract according to the diet, at phylum and genus level. Comparisons in the ST are omitted due to the higher percentages of plant-derived sequences, especially in fish fed AA0 (Table 3).

**Table 3:** Percentages of Chloroplast, Algae, Mitochondria and Bacterial Sequences in all the pools of different sections and tanks. ST: Stomach, FG: Foregut, MG: Midgut, HG: Hindgut

	N. of sequences	% Chloroplast sequences	% Algae sequences	% Mitochondria sequences	% Bacterial sequences	N. of bacterial sequences
**FM100**						
**ST1**	3727	32.28	0.00	3.17	64.56	2406
**ST2**	3670	6.29	0.00	5.61	88.09	3233
**ST3**	2646	0.04	0.00	0.00	99.96	2645
**ST**		12.87	0.00	2.93	84.20	
**FG 1**	3568	3.00	0.00	0.95	96.05	3427
**FG 2**	7338	5.04	0.00	11.37	83.59	6134
**FG 3**	4689	10.11	0.13	20.54	69.23	3246
**FG**		6.05	0.04	10.95	82.96	
**MG 1**	3721	0.56	0.00	0.13	99.30	3695
**MG 2**	4334	1.15	0.00	0.48	98.36	4263
**MG 3**	4065	10.11	0.00	10.75	79.14	3217
**MG**		3.94	0.00	3.79	92.27	
**HG 1**	3926	0.36	0.00	0.03	99.62	3911
**HG 2**	3661	12.48	0.00	9.31	78.20	2863
**HG 3**	3499	5.06	0.00	6.23	88.71	3104
**HG**		5.97	0.00	5.19	88.84	
**AA0**						
**ST1**	2633	48.20	0.00	5.32	46.49	1224
**ST2**	4519	84.42	0.00	8.48	7.10	321
**ST3**	3995	88.39	0.00	9.01	2.60	104
**ST**		73.67	0.00	7.60	18.73	
**FG 1**	2573	17.61	0.04	1.44	80.92	2082
**FG 2**	3579	18.75	0.00	1.45	79.80	2856
**FG 3**	3810	4.17	0.05	0.16	95.62	3643
**FG**		13.51	0.03	1.02	85.44	
**MG 1**	3386	2.84	13.94	0.74	82.49	2793
**MG 2**	2693	10.36	0.00	0.93	88.71	2389
**MG 3**	3633	5.53	1.40	0.41	92.65	3366
**MG**		6.24	5.11	0.69	87.95	
**HG 1**	3015	3.78	0.10	0.17	95.95	2893
**HG 2**	2890	9.03	0.07	0.87	90.03	2602
**HG 3**	2688	6.96	0.00	0.45	92.60	2489
**HG**		6.59	0.06	0.49	92.86	
**WATER**	3281	0.00	0.24	0.00	99.76	3273

In the FG, the presence of *Proteobacteria* was more relevant in AA0 samples than in FM100 (36.2% vs 13.4%, p-value = 0.222), while *Firmicutes* were more abundant in FM100 than in AA0 (43.1% vs 22.8%, p-value = 0.090). *Streptococcus* genus abundance was higher in the FM100 group than in AA0 (23.4% vs 4.7%, p-value = 0.097), and fishmeal replacement also affected negatively the genera *Staphylococcus* and *Lactobacillus* throughout the gut, including the FG (3.3% vs 2.1%, p-value = 0.422 and 2.7% vs 1.1%, p-value = 0.099, respectively). In contrast, the genus Finegoldia was present in lower proportions in FM100 than in AA0 (1.3% vs 3.6%, p-value = 0.300). The genera *Photobacterium* (8.7% vs 0.1%, p-value = 0.364), *Enhydrobacter* (7.0% vs 0.4%, p-value = 0.339), *Sphingomonas* (5.1% vs 0.3%, p-value = 0.242) and an UG of the family Enterobacteriaceae (6.9% vs 0.6%, p-value = 0.331) were more highly represented in the FG of fish fed the plant-based diet than in FM100.

At phylum level, differences between the FM100 group and the AA0 group, in abundance of Firmicutes (36.2% vs 31.2%, p-value = 0.361) and Proteobacteria (21.90% vs 29.2%, p-value = 0.501), decreased in the MG, although Bacteroidetes were more abundant in fish fed the fishmeal diet than in fish fed the vegetal diet (11.0% vs 2.4%, respectively, p-value = 0.279). At genus level, *Bacteroides* were highly represented in the MG of fish fed FM100 than in AA0 (8.5% vs 0.5%, p-value = 0.268), such as *Lactobacillus* (11.0% vs 3.2%, p-value = 0.251), *Megamonas* (3.7% vs 1.9%, p-value = 0.436) and *Staphylococcus* (3.4% vs 0.9%, p-value = 0.263). The most abundant genera of the phylum Firmicutes in animals receiving the vegetable diet were *Streptococcus* (7.8%, vs 5.5% in fish fed FM100, p-value = 0.363) and *Proteiniclasticum* (7.6%), observed exclusively in this group of fish (p-value = 0.372). Among Proteobacteria, Enterobacteriaceae (7.0%), and Legionellaceae (3.8%) were the most represented families in this section in fish fed FM100, while in AA0 samples of the MG the most common genus of Proteobacteria was *Photobacterium* (8.8%), followed by *Pseudomonas* (5.3%) and an UG of Rhodobacteraceae (3.0%).

A significantly higher presence of *Proteobacteria* (47.9% vs 15.5%, p-value = 0.022) and lower abundances of *Firmicutes* (17.6% vs 29.9%, p-value = 0.079) and *Actinobacteria* (26.2% vs 46.9%, p-value = 0.092) were observed in the HG of fish fed the plant-based meal diet compared to FM100. An UG of the family Micrococcaceae was exclusively observed in fish fed FM100 (8.6%, p-value = 0.269), although as stated above, *Corynebacterium* and *Propionibacterium* were the most abundant genera belonging to this phylum in fish fed both diets. *Streptococcus* was the major taxon among the *Firmicutes* genera in the FM100 group, being significantly overrepresented compared to the AA0 samples (12.7% vs 3.7%, p-value = 0.009), followed by *Staphylococcus* (2.6% vs 0.8%, p-value = 0.232) and *Lactobacillus* (2.2% vs 1.8%, p-value = 0.723). *Proteiniclasticum* (2.4%) was only found in fish fed the vegetable diet, as occurred in the MG (p-value = 0.365). The *photobacterium* was very abundant and exclusively observed in the HG of fish fed AA0 (25.4%, p-value = 0.103), and the above-mentioned UG of the family *Rhodobacteraceae* was also abundant in this group of fish (8.0%, vs 0.2% in the FM100 group, p-value = 0.230). In fish fed FM100, an UG of the family *Legionellaceae* was the most observed genus belonging to the phylum Proteobacteria (6.3%, vs 2.0 in fish fed AA0, p-value = 0.519).

**Fig 8:**
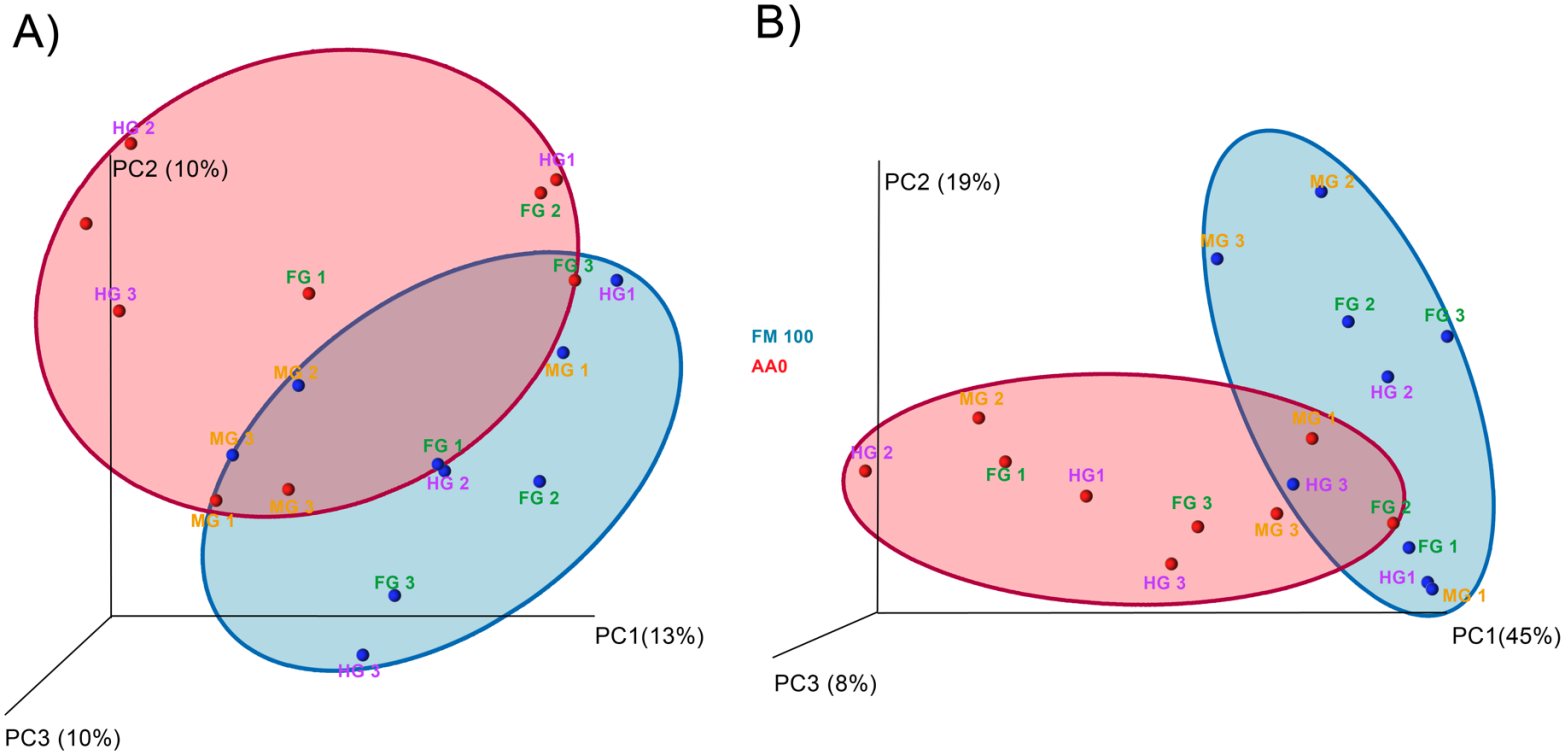
Principal coordinates analysis (PCoA) of Unweighted (A) and Weighted (B) Unifrac distances of microbial communities associated to the gut, according to diet. A beta diversity distance matrix was computed from the previously constructed OTU table using UniFrac analysis. Unweighted (presence/absence matrix) and weighted (presence/absence/abundance matrix) UniFrac distances were used to construct the PCoA plots. Circles in red and blue represent different gut section of fish fed AA0 and FM100, respectively.

Finally, unweighted and weighted PCoA showed a certain differentiation in the microbiota associated to the gut of fish fed FM100 and AA0 (Fig 8). Unweighted PCoA (PC1 = 13%, PC2 = 10%, PC3 = 10%) grouped samples by diet. A higher separation between different sections of the gut of fish fed AA0 was observed in comparison with FM100 samples, although two outlier samples corresponding to FM100 were found. Separation among diets was clearer in the weighted PCoA (PC1 = 45%, PC2 = 19%, PC3 = 8%). First Component grouped different sections of the intestine of fish fed fishmeal, while gut sections of fish fed the vegetable diet appeared more separated from each other along the X axis. Second Component of the PCoA had the opposite effect on samples, grouping AA0 gut sections and separating FM100 ones.

## Discussion

### Gastrointestinal microbiota of gilthead sea bream

Although Microbiota composition seems to differ among fish species, in general terms, fish harbour a microbiota that is dominated mainly by the phyla *Proteobacteria*, *Firmicutes* and *Actinobacteria* [37].All fish gut samples in this study and most fish gut samples in previous studies shared *Proteobacteria* and *Firmicutes* as the most dominant phyla [27,38,39]. *Actinobacteria* were found in our samples and also in grass carp [38] and rainbow trout [39]. Nevertheless, *Bacteroidetes* [30] and *Fusobacteria* [28] were the most representative phyla in seabass and carp, respectively. At genus level, *Achromobacter*, *Acinetobacter*, *Aeromonas*, *Alcaligenes*, *Alteromonas*, *Bacteroides*, *Corynebacterium*, *Clostridium*, *Cytophaga*, *Flavobacterium*, *Micrococcus*, *Moraxella*, *Photobacterium*, *Pseudomonas* and *Vibrio* have been described as the most common genera retrieved in marine fish, as well as different genera of lactic acid bacteria (*Streptococcus*, *Lactobacillus*, *Leuconostoc*, *Carnobacterium*), belonging to the phylum Firmicutes [10,22,25,27].

According to our own results, most of these genera were present in the GIT of gilthead sea bream. DNA extraction was performed on gastrointestinal content samples, obtained from the gut after scraping the mucosa, thus genera represented both, luminal (allochthonous) and mucosal communities (autochthonous).

Results were consistent with results obtained on the microbiota present in the stomach and the gut in sea bream with other methods [13,26]. However, in a previous study using tag pyrosequencing [31], Proteobacteria, Firmicutes, Actinobacteria and Bacteroidetes were the most abundant phyla observed in the gut, whilst Diaphorobacter (belonging to β-Proteobacteria) was the dominant genus in all fish examined. This finding could be explained by the fact that bacterial DNA was extracted from the gut tissue, whereas the majority of previous works had analysed the microbiota from the intestinal content [40].

In the present work, the analysis of the microbiota of the stomach was limited by chloroplast and mitochondria sequence contamination, which has been found in 16S rRNA gene analysis when using 454 pyrosequencing for the analysis of microbial communities in plants and folivorous arthropods [41]. This technical limitation, intrinsic to 16S rDNA sequencing, must be born in mind. Chloroplast and mitochondria sequences were ruled out for further quantitative analysis, although it also eliminated Cyanobacteria and *Mitochondria* (Rikettsiales), thus, introducing a population bias, mainly in the ST samples where aquatic bacteria (Cyanobacteria) could be highly represented, while Rickettsiales are pathogenic or endosymbionts not expected in the stomach. Relative abundance of *Chloroplasts and Mitochondria* was much higher in the stomach, especially in fish fed the AA0 diet, compared with gut sections (always below 20% and 10% for diets AA0 and FM100, respectively) and the differences between the diets disappeared when moving through the GIT, evidencing that they must predominantly correspond to vegetable components of the diet that are degraded during digestion. In addition, the lower microbial diversity found in the stomach could also be due to the restrictive environmental conditions found in the ST, as occurs in most vertebrates.

### Recirculating saltwater system microbiota

In the present study, the microbiota of the Recirculating Aquaculture System (RAS) was dominated by different genera of the family *Flavobacteriaceae*, widely distributed in diverse habitats, including marine environments, in which they may be numerically dominant [42]. The family *Rhodobacteraceae*, which often occurs in aquatic habitats, was abundantly observed in other RAS [43], and *Saprospiraceae*, which was also well-represented, was found to be linked with activated sludge [44] and its presence could be due to the high content of organic matter in the system. Uptake of RAS water by marine fish takes place continuously, hence GIT microbiota is expected to be a mixture of autochthonous and allocthonous bacteria [45]. Nevertheless, in our study, RAS had a different microbiota composition and greater diversity of that observed in the gilthead sea bream GIT. *Flavobacteriaceae* and *Saprospiraceae* were underrepresented in all sections compared to water, while, in comparison, *Rhodobacteraceae* was found in higher percentages in the whole digestive tract, particularly in the HG of AA0, suggesting the diet could affect the colonization of GIT by bacteria present in the surrounding water. Further studies should be performed in order to clarify the origin of different bacterial groups and their capacity to colonize the GIT of fish.

The microbial community of Recirculating Aquaculture Systems (RAS) is influenced by several factors, such as feed type, feeding regime, management routines, variation in system design, water composition parameters [46] and the selective pressure of biofilters [47]. In addition, each fish species introduces its own microbiota of skin, gills and GIT [48]; make-up water also alters its original microbial composition, and fish feed, equipment used in and about the system and staff/visitors in contact [49] may also introduce different taxa of bacteria. Moreover, storage and processing of samples and PCR efficiency can affect the presence and relative abundance of different taxa [30, 50]. Hence, microbial diversity and composition in RAS water varies from one system to another, making comparisons difficult [51].

### Impact of fishmeal replacement

Total fishmeal replacement by plant protein concentrate has been reported in sea bream juveniles [8] with positive growth and nutrient efficiency. The success of total fishmeal substitution was due to the high digestibility of the protein source concentrate and also to the balanced dietary amino acid profile.

In our study, no significant differences in terms of growth and nutritive parameters could be found between diets. However, significant differences were observed in the survival rate. Mortality did not seem to be associated with any specific pathology. The presence of high non starch polysaccharide and other antinutrients substances, as tannins, in the AA0 diet, may cause a decrease in the availability of nutrients, including amino acids, producing imbalances with the direct consequence on immune organs and responses [52].

Present study does not include data of microbial community before the feeding trial. Fish were obtained from an aquaculture farm, so ‘initial’ microbiota would be already influenced by artificial diets, differing from wild gut microbiota and providing little information about changes in the gut community composition or abundance. In addition, present work is focused on determine the differences in the microbial pattern in response to different experimental diets.

In STs of fish fed FM100, the genera *Corynebacterium*, *Propionibacterium* and *Clostridium* were the most abundant, while the family *Enterobacteriaceae*, was most abundant in fish fed the vegetable diet. In fact, the latter organisms are regarded as efficient secretors of polysaccharide hydrolases [53,54], being compatible with the higher fibre content of AA0.

Fishmeal replacement did not induce significant changes in microbial richness throughout the gut, as no significant differences in Alpha diversity Indices, Observed Species and Phylogenetic Distance were determined between the two groups of fish, which is in agreement with previous reports [26]. Other studies in rainbow trout reported higher bacterial richness [29], lower microbial diversity [55] or only minor changes in the microbiota composition [56] with different levels of substitution, while contradictory effects of fishmeal replacement on the microbial diversity were observed in Atlantic salmon [57,58].

Fishmeal replacement had a negative effect on the relative abundance of *Firmicutes* throughout the gut, particularly on the genera *Streptococcus* and *Lactobacillus*, which are lactic acid bacteria. These are prevalent constituents of the intestinal microbiome of many fish species and are generally considered beneficial organisms associated with a healthy intestinal epithelium [59], and some strains of these taxa can inhibit adhesion of several fish pathogens, ensuring the maintenance of a balanced microbiota, which is crucial in the prevention of diseases, especially GIT infections [60]. On the other hand, fish fed the vegetable mixture diet exhibited a higher percentage of *Proteobacteria* along the whole digestive tract. The genus *Photobacterium* was highly represented in all sections of the gut of fish fed AA0, particularly in the HG. Some species of the genus *Photobacterium* are secondary pathogens of marine life and its great abundance in the GIT of this group of fish might suggest an alteration of gut immune mechanisms of gilthead sea bream (they could be also primary pathogens, but no clinical signs associated to any pathology were observed). Nevertheless, differences in *Photobacterium* abundance could also be explained by differences in fibre and NSP between FM100 and AA0, as this genus has also been previously reported to degrade cellulose [53,54]. The genus *Pseudomonas*, which also has cellulolytic activity, was slightly more abundant throughout the GIT of AA0, especially in the MG.

It is likely that low or moderate fishmeal substitutions in sea bream feeds do not have a significant effect, or even have a positive effect, on growth [61] and also on microbiota composition and diversity [7], preventing the establishment of an evident dietary effect [26]. Nevertheless, high fishmeal replacement could produce alterations in the non-specific immune system in gilthead sea bream [6], which could be the main reason of the higher mortality in the group of fish fed the AA0 diet. Soybean protein has been reported to induce enteropathy in salmonids and others fish species [62], with a variety of intestinal disturbances including increased permeability [63] promoting inflammatory secretions that lead to greater immune sensitivity [64,65]. In our work, changes in the microbial patterns detected in the vegetable diet, particularly in the immune-competent segments of the hindgut, could also render fish prone to infection. An imbalanced microbiota could alter immune regulatory functions of the gut and contribute to the development of diseases [13], particularly if Proteobacteria are the dominant clade, which includes potential pathogen genera (such as *Pseudomonas* and *Photobacterium*). This is in agreement with the observed susceptibility to infection of Atlantic salmon fed soybean, showing high levels of lysozyme and IgM in the mid- and distal-intestinal mucosa and an elevated gut inflammatory response [66].

A core microbiota has been suggested in different species [27,39,56], however, large individual variations within fish with a similar genetic background, fed the same diet and maintained under the same environmental conditions, have been described in previous reports [26,29,55] probably due to a strong host genotype influence on the bacterial composition [39,58]. In the present study, PCoA showed that gut content samples from fish that followed the same diet clustered together, although the AA0 diet showed greater dispersion. Hence, endogenous and exogenous factors but also the great variability of sources and proportions of ingredients used in feeds can modify the microbiome constitution. The link between diet and gut microbiota and the related changes in gut morphology and the immune system should be subject to further investigation, in order to understand the greater mortality observed as a consequence of the vegetable diet.

In conclusion, our study revealed that the total fishmeal replacement in diets for gilthead sea bream was nutritionally satisfactory and introduced no change in the total microbial diversity or richness, but altered the GIT microbiota profile at HG level, being a GIT section rich in immune cells. There was also an increase in the mortality rate. Further studies will determine if the adverse effect observed, possibly at immune level, was due to vegetable components of the diet or if it was the consequence of the microbial imbalance that they caused, or both. Development of new diets with new sources of ingredients, and possibly probiotics, will help in these investigations that constitute the keystone to the development of more efficient, economic and sustainable feeds in aquaculture.

## Supporting Information

S1 Fig Rarefaction curves (Chao1 index) showing the microbial community complexes in the different gut sections of gilthead sea bream fed different experimental diets (AA0 and FM100). (TIF)

S1 File ARRIVE Guidelines Checklist. (DOC)
