# Supplementary figures and images for: Impact of Fishmeal Replacement in Diets for Gilthead Sea Bream (Sparus aurata) on the Gastrointestinal Microbiota Determined by Pyrosequencing the 16S rRNA Gene
Source: PLoS One. 2015 Aug 28;10(8):e0136389. doi: 10.1371/journal.pone.0136389 (PMC4552794; doi:10.1371/journal.pone.0136389)

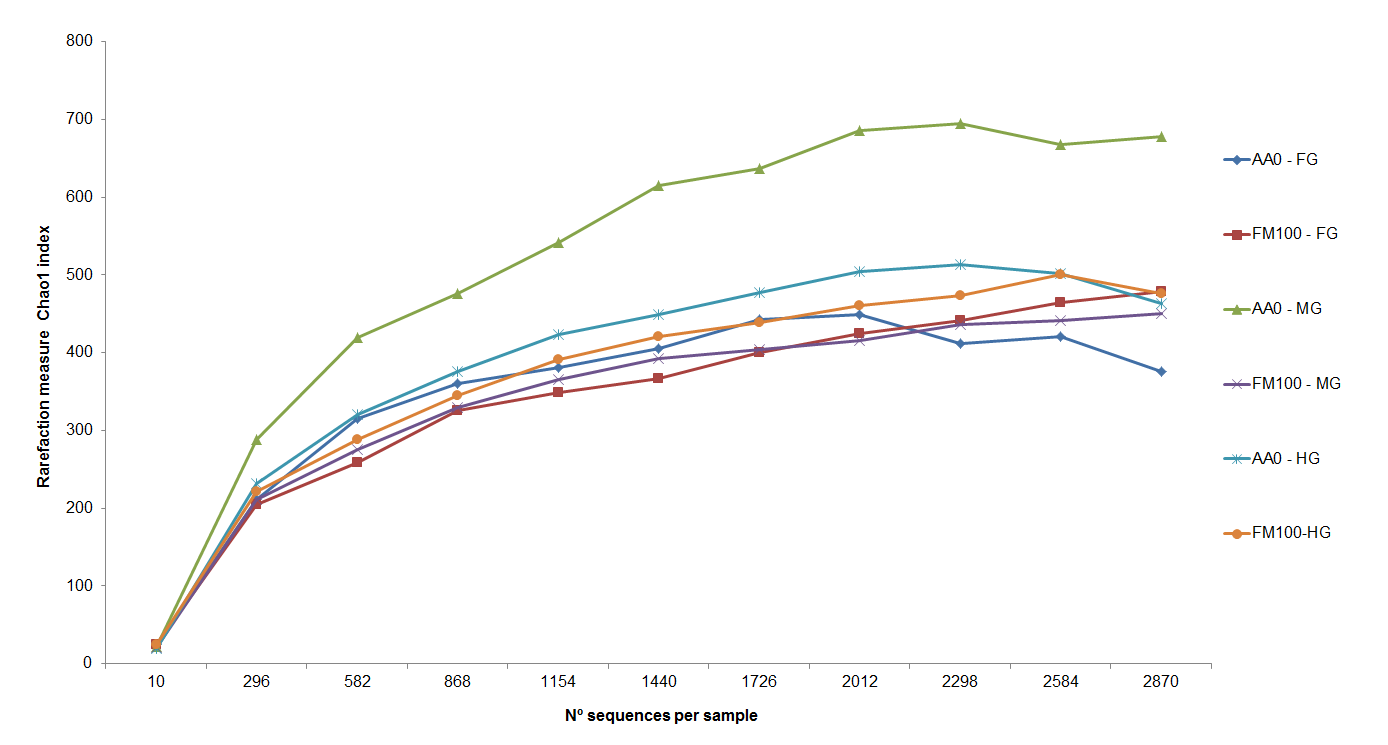

Supplement: S1 Fig — (TIF) [file pone.0136389.s001.tif]
